# Graph neural networks based log anomaly detection and explanation

**DOI:** 10.1007/s10618-026-01235-6

**Published:** 2026-07-03

**Authors:** Zhong Li, Jiayang Shi, Matthijs van Leeuwen

**Affiliations:** 1 Leiden Institute of Advanced Computer Science (LIACS), Leiden University, Leiden, The Netherlands; 2 The Intelligent Computing Research Center, Great Bay University, Dongguan, China

**Keywords:** Log analysis, Log anomaly detection, Graph neural networks, Knowledge discovery from graphs

## Abstract

Event logs are widely used to record the status of high-tech systems, making log anomaly detection important for monitoring those systems. Most existing log anomaly detection methods take a log event count matrix or log event sequences as input, exploiting quantitative and/or sequential relationships between log events to detect anomalies. However, only considering quantitative or sequential relationships may result in low detection accuracy. To alleviate this problem, we propose a graph-based method for unsupervised log anomaly detection, dubbed *Logs2Graphs*, which first converts event logs into attributed, directed, and weighted graphs, and then leverages graph neural networks to perform graph-level anomaly detection. Specifically, we introduce One-Class Digraph Inception Convolutional Networks, abbreviated as OCDiGCN, a novel graph neural network model for detecting graph-level anomalies in a collection of attributed, directed, and weighted graphs. By integrating graph representation and anomaly detection, OCDiGCN learns a specialized representation that leads to high detection accuracy. Crucially, we furnish a concise set of nodes pivotal in OCDiGCN’s prediction as explanations for each detected anomaly, offering valuable insights for subsequent root cause analysis. Experiments on five benchmark datasets show that *Logs2Graphs* exhibits comparable or superior performance when compared to state-of-the-art log anomaly detection methods.

## Introduction

Modern high-tech systems, such as cloud computers or lithography machines, typically consist of a large number of components. Over time these systems have become larger and more complex, making manual system operation and maintenance hard or even infeasible (Li and van Leeuwen 2022). Therefore, automated system operation and maintenance is highly desirable. To achieve this, system logs are universally used to record system states and important events. By analyzing these logs, faults and potential risks can be identified, and remedial actions may be taken to prevent severe problems. System logs are usually semi-structured texts though, and identifying anomalies through log anomaly detection is often challenging.

Since both industry and academia show great interest in identifying anomalies from logs, a plethora of log anomaly detection methods have been proposed. Existing log anomaly detection methods can be roughly divided into three categories: quantitative-based, sequence-based, and graph-based methods. Specifically, quantitative-based methods, such as OCSVM (Schölkopf et al. 2001) and PCA (Xu et al. 2009), utilize a log event count matrix to detect anomalies, and are therefore unable to capture semantic information of and sequential information between log events. Meanwhile, sequence-based methods, including DeepLog (Du et al. 2017) and LogAnomaly (Meng et al. 2019), aim to detect anomalies by taking sequential (and sometimes semantic) information into account. They cannot consider the full structure among log events though. In contrast, graph-based methods, such as GLAD-PAW (Wan et al. 2021) and GLAD (Li et al. 2023), convert logs to graphs and exploit semantic information as well as the structure among log events, exhibiting the following three advantages over the former two categories of methods (Jia et al. 2017): (1) they are able to identify problems for which the structure among events is crucial, such as performance degradation; (2) they are capable of providing contextual log messages corresponding to the identified problems; and (3) they can provide the ‘normal’ operation process in the form of a graph, helping end-users find root-causes and take remedial actions. However, graph-based methods like GLAD transform log events into *undirected* graphs though, which may fail to capture important information on the order among log events. Moreover, most existing graph-based methods perform graph representation and anomaly detection separately, leading to suboptimal detection accuracy.

Moreover, as highlighted by Li et al. (2023), with the growing adoption of anomaly detection algorithms in safety-critical domains, there is a rising demand for providing explanations for the decisions made within those domains. This requirement, driven by ethical considerations and regulatory mandates, underscores the importance of accountability and transparency in such contexts. Moreover, in practical applications, the attainment of precise anomaly explanations contributes to the timely isolation and diagnosis of anomalies, which can mitigate the impact of anomalies by facilitating early intervention (Yang et al. 2021b). However, to our knowledge, most existing log anomaly detection methods focus exclusively on accurate detection without giving explanations.

To overcome these limitations, we propose *Logs2Graphs*, a graph-based unsupervised log anomaly detection approach for which we design a novel one-class graph neural network. Specifically, *Logs2Graphs* first utilizes off-the-shelf methods to learn a semantic embedding for each log event, and then assigns log messages to different groups. Second, *Logs2Graphs* converts each group of log messages into an attributed, directed, and weighted graph, with each node representing a log event, the node attributes containing its semantic embedding, directed edges representing how an event is followed by other events, and the corresponding edge weights indicating the number of times the events follow each other. Third, by coupling the graph representation learning and anomaly detection objectives, we introduce One-Class Digraph Inception Convolutional Networks (OCDiGCN) as a novel method to detect anomalous graphs from a set of graphs. As a result, *Logs2Graphs* leverages the rich and expressive power of attributed, directed, and edge-weighted graphs to represent logs, followed by using graph neural networks to effectively detect graph-level anomalies, taking into account both semantic information of log events and structure information (including sequential information as a special case) among log events. Importantly, by decomposing the anomaly score of a graph into individual nodes and visualizing these nodes based on their contributions, we provide understandable explanations for identified anomalies.

Overall, our contributions can be summarized as follows: (1) We introduce *Logs2Graphs*, which *recasts log anomaly detection as unsupervised graph-level anomaly detection* and represents log groups as attributed, directed, and edge-weighted graphs, thereby enabling quantitative, sequential, and richer structural anomalies to be analyzed within one unified representation; (2) We introduce OCDiGCN, a general end-to-end unsupervised graph-level anomaly detection method for attributed, directed and edge-weighted graphs. By coupling the graph representation and anomaly detection objectives, we improve the potential for accurate anomaly detection over existing approaches; (3) For each detected anomaly, we identify important nodes as explanations, offering cues for subsequent root cause diagnosis; (4) We empirically compare our approach to eight state-of-the-art log anomaly detection methods on five benchmark datasets, showing that *Logs2Graphs* performs at least on par with and often better than its competitors.

**Comparison with our previous work**. This paper has been accepted as a two-pages extended abstract (Li et al. 2024c) in ICSE 2024, in which we only presented the problem addressed, a brief summary of the algorithm, and the achieved main results. In contrast, in this full paper we give the motivations of designing algorithms, present related work and the design details of algorithms, elucidate the experiment setting, and present full experiment results with detailed interpretation.

**Organization of the paper.** The remainder of this paper is organized as follows. Section 2 revisits related work, after which Sect. 3 formalizes the problem. Section 4 describes Digraph Inception Convolutional Networks (Tong et al. 2020a), which are used for *Logs2Graphs* in Sect. 5. We then evaluate *Logs2Graphs* in Sect. 6 with publicly available datasets. We conclude this paper in Sect. 8.

## Related work

Graph-based log anomaly detection methods usually comprise five steps: log parsing, log grouping, graph construction, graph representation learning, and anomaly detection. In this paper we focus on graph representation learning, log anomaly detection, and explanation, thus only revisiting related work in these fields.

### Graph representation learning

Graph-level representation learning methods, such as GIN (Xu et al. 2018) and Graph2Vec (Narayanan et al. 2017), are able to learn a mapping from graphs to vectors. Further, graph kernel methods, including Weisfeiler-Lehman (WL) (Shervashidze et al. 2011) and Propagation Kernels (PK) (Neumann et al. 2016), can directly provide pairwise distances between graphs. Both types of methods can be combined with off-the-shelf anomaly detectors, such as OCSVM (Schölkopf et al. 2001) and iForest (Liu et al. 2008), to perform graph-level anomaly detection.

To improve on these naïve approaches, efforts have been made to develop graph representation learning methods especially for anomaly detection. For instance, OCGIN (Zhao and Akoglu 2021) and GLAM (Zhao et al. 2022) combine the GIN (Xu et al. 2018) representation learning objective with the SVDD objective (Tax and Duin 2004) to perform graph-level representation learning and anomaly detection in an end-to-end manner. GLocalKD (Ma et al. 2022) performs random distillation of graph and node representations to learn ‘normal’ graph patterns. Further, OCGTL (Qiu et al. 2022) combines neural transformation learning and one-class classification to learn graph representations for anomaly detection. Besides, ARMET (Li et al. 2024b) aims to identify anomalous graphs from a set of unlabeled graphs by using easily accessible normal graphs from a different but related domain. Although these methods are unsupervised or semi-supervised, they can only deal with attributed, undirected, and unweighted graphs.

iGAD (Zhang et al. 2022b) considers graph-level anomaly detection as a graph classification problem and combines attribute-aware graph convolution and substructure-aware deep random walks to learn graph representations. However, iGAD is a supervised method, and can only handle attributed, undirected, and unweighted graphs. CODEtect (Nguyen et al. 2023) takes a pattern-based modeling approach using the minimum description length principle and identifies anomalous graphs based on *motifs*. CODEtect can (only) deal with labeled, directed, and edge-weighted graphs, but is computationally very expensive. In contrast, we introduce a general unsupervised method for graph-level anomaly detection that can handle attributed, directed and edge-weighted graphs.

Moroever, several studies have explored *one-class* graph neural networks for anomaly detection at the node level. Wang et al. (2021) propose one-class graph neural networks for attributed networks by jointly learning node representations and a hypersphere that encloses normal nodes. Similarly, Zhou et al. (2021) introduce subtractive aggregation to model node abnormality as deviation from local neighborhoods and optimize a hypersphere-based objective for attributed network anomaly detection. These methods demonstrate the effectiveness of combining graph representation learning with one-class classification objectives, but they focus on detecting anomalous *nodes* in attributed, undirected networks.

### Log anomaly detection and explanation

Log anomaly detection methods can be roughly divided into: (1) traditional, ‘shallow’ methods, such as principal component analysis (PCA) (Xu et al. 2009), one-class SVM (OCSVM) (Schölkopf et al. 2001), isolation forest (iForest) (Liu et al. 2008), and histogram-based outlier score (HBOS) (Goldstein and Dengel 2012), which take a log event count matrix as input and analyze quantitative relationships; (2) deep learning based methods, such as DeepLog (Du et al. 2017), LogAnomaly (Meng et al. 2019), AutoEncoder (Farzad and Gulliver 2020), and transformer-based models such as LogBERT (Guo et al. 2021), which employ sequences of log events (and sometimes their semantic embeddings) as input, analyzing sequential information and possibly semantic information of log events to identify anomalies; and (3) graph-based methods, such as TCFG (Jia et al. 2017) and GLAD-PAW (Wan et al. 2021), which first convert logs into graphs and then perform graph-level anomaly detection.

To our knowledge, at the time of the original study design in early 2024, only a few studies (Wan et al. 2021; Zhang et al. 2022a; Xie et al. 2022; Li et al. 2023) had explored the use of graph neural networks for log anomaly detection. GLAD (Li et al. 2023) transforms logs into undirected, weighted, and attributed heterogeneous graphs and proposes a temporal-attentive graph edge anomaly detection model to detect anomalous relations. However, converting logs into undirected graphs may result in loss of important sequential information. DeepTraLog (Zhang et al. 2022a) combines traces and logs to generate a so-called Trace Event Graph, which is attributed and directed; on this basis, it trains a Gated Graph Neural Networks based Deep Support Vector Data Description model to identify anomalies. However, its approach requires the availability of both traces and logs, and is unable to handle edge weights. GLAD-PAW (Wan et al. 2021) formulates anomaly detection through next-event prediction on weighted directed session graphs, whereas LogGD (Xie et al. 2022) performs supervised graph classification and therefore requires labeled normal and anomalous training data. In contrast, *Logs2Graphs* recasts log anomaly detection as *unsupervised graph-level anomaly detection* on attributed, directed, and edge-weighted graphs, couples representation learning and one-class detection in an end-to-end manner, and provides anomaly explanations aligned with the anomaly score. These differences clarify that our contribution is not merely “unsupervised + explainable”, but a distinct formulation and end-to-end treatment of log anomaly detection on expressive directed log graphs.

Beyond anomaly detection, several studies have investigated modeling dependencies among log events to support system-level understanding and diagnosis. For example, LADDERS (Mondal et al. 2024) integrates anomaly scoring with causal discovery to identify dependencies across heterogeneous subsystems and facilitate root cause analysis in enterprise environments. Relatedly, recent advances in high-dimensional causal discovery (Mondal et al. 2025) focus on scalable inference of dependency structures from observational data. While these approaches primarily target diagnosis and causal analysis rather than representation learning for anomaly detection, they highlight the importance of capturing inter-event dependencies in complex systems.

Since the initial public release of our work, including a preliminary version in the *ICSE 2024 Companion Proceedings* (Li et al. 2024c) and an openly released technical report and implementation, the broader log anomaly detection literature has expanded rapidly, as also reflected in recent comprehensive empirical studies (Ali et al. 2025). Given this rapid growth, the discussion below focuses on *closely related graph-based follow-up work* rather than attempting to re-survey the entire log anomaly detection field. Closely related post-2024 examples include LogGT (Wang et al. 2024), which models cross-system log sequences as heterogeneous graphs and combines graph transformers with transfer learning; IST-GCN (Xu and Li 2024), which jointly models temporal and event-similarity relations through directed and undirected graphs while emphasizing interpretability; RTGNN (Han et al. 2026), which introduces temporal event graphs and reinforcement learning for class-imbalanced log anomaly detection; and BertGCN-style raw-log graph modeling (Alsalmi et al. 2026). These studies reinforce the continued interest in graph-based log modeling, while also exploring complementary settings such as heterogeneous graphs, dual spatial-temporal graphs, temporal event graphs, cross-system transfer, and raw-log graph construction. We discuss them here to position the paper against the current literature as of 2026, but do not add them retrospectively to the original benchmark comparison because they postdate the original experimental study design reported in this manuscript.

Although anomaly explanation has received much attention in traditional anomaly detection (Li et al. 2023; Li and Van Leeuwen 2023), only a few studies (Yang et al. 2021a) considered log anomaly explanation. Specifically, PLELog (Yang et al. 2021a) offers explanations by quantifying the significance of individual log events within an anomalous log sequence, thereby facilitating improved identification of relevant log events by operators. Similarly, our method provides explanations for anomalous log groups by identifying and visualising a small subset of important nodes.

## Problem statement

Before we state the log anomaly detection problem, we first introduce notation and definitions regarding event logs and graphs.

**Event logs**. *Logs* are used to record system status and important events, and are usually collected and stored centrally as log files. A *log file* typically consists of many *log messages*. Each *log message* is composed of three components: a timestamp, an event type (*log event* or *log template*), and additional information (*log parameters*). *Log parsers* are used to extract log events from log messages.

Further, log messages can be grouped into *log groups* (a.k.a. *log sequences*) using certain criteria. Specifically, if a *log identifier* is available for each log message, one can group log messages based on such identifiers. Otherwise, one can use a *fixed* or *sliding window* to group log messages. Besides, counting the occurrences of each log event within a log group yields an *event count vector*. Consequently, for a log file consisting of many log groups, one can obtain an *event count matrix*. The process of generating an *event count matrix* (or other feature matrix) is known as *feature extraction*. Extracted features are often used as input to an anomaly detection algorithm to identify *log anomalies*, i.e., log messages or log groups that deviate from what is considered ‘normal’.

In this work, “normal” behavior is defined with respect to the training data. That is, any operational setting (e.g., workload characteristics or usage patterns) that is sufficiently represented in the training set can be learned as normal and will not be regarded as anomalous. For clarity of presentation, we focus on the common setting where the training data corresponds to a single system under normal operation with a relatively stable workload, but the proposed formulation is not inherently restricted to this setting.

**Graphs**. We consider an attributed, directed, and edge-weighted graph $\mathcal {G} = (\mathcal {V}, \mathcal {E}, {\mathbf {X}}, {\mathbf {Y}})$, where $\mathcal {V} = \{v_{1},...,v_{|\mathcal {V}|}\}$ denotes the set of *nodes* and $\mathcal {E} = \{e_{1},...,e_{|\mathcal {E}|}\} \subseteq \mathcal {V}\times \mathcal {V}$ represents the set of edges. If $(v_{i},v_{j})\in \mathcal {E}$, then there is an edge from node $v_{i}$ to node $v_{j}$. Moreover, ${\mathbf {X}} \in \mathbb {R}^{|\mathcal {V}|\times d}$ is the node attribute matrix, with the *i*-th row representing the attributes of node $v_{i}$, and *d* is the number of attributes. Besides, ${\mathbf {Y}} \in \mathbb {N}^{|\mathcal {E}|\times |\mathcal {E}|}$ is the edge-weight matrix, where ${\mathbf {Y}}_{ij}$ represents the weight of the edge from node $v_{i}$ to node $v_{j}$.

Equivalently, $\mathcal {G}$ can be described as $({\mathbf {A}}, {\mathbf {X}}, {\mathbf {Y}})$, with adjacency matrix ${\mathbf {A}} \in \mathbb {R}^{|\mathcal {V}|\times |\mathcal {V}|}$, where ${\mathbf {A}}_{ij} = \mathbb {I}[(v_{i},v_{j}) \in \mathcal {E}]$ indicates whether there is an edge from node $v_{i}$ to node $v_{j}$, for $i,j \in \{1,...,|\mathcal {V}|\}$.

### Graph-based log anomaly detection

Given a set of log files, we let $\mathcal {L} = \{L_{1},...,L_{|\mathcal {L}|}\}$ denote the set of unique log events. We divide the log messages into *M* log groups ${\mathbf {Q}} = \{ {\mathbf {q}}_{1},...,{\mathbf {q}}_{m},...,{\mathbf {q}}_{M}\}$, where ${\mathbf {q}}_{m}=\{{\mathbf {q}}_{m1},...,{\mathbf {q}}_{mn},...,{\mathbf {q}}_{mN}\}$ is a log group and ${\mathbf {q}}_{mn}$ a log message.

For each log group ${\mathbf {q}}_{m}$, we construct an attributed, directed, and edge-weighted graph $\mathcal {G}_{m} = (\mathcal {V}_{m}, \mathcal {E}_{m}, {\mathbf {X}}_{m}, {\mathbf {Y}}_{m})$ to represent the log messages and their relationships. Specifically, each node $v_{i} \in \mathcal {V}_{m}$ corresponds to exactly one log event $L \in \mathcal {L}$ (and vice versa). Further, an edge $e_{ij} \in \mathcal {E}_{m}$ indicates that log event *i* is at least once immediately followed by log event *j* in ${\mathbf {q}}_{m}$. Attributes ${\mathbf {x}}_{i} \in {\mathbf {X}}_{m}$ represent the semantic embedding of log event *i*, and $y_{ij} \in {\mathbf {Y}}_{m}$ is the weight of edge $e_{ij}$, representing the number of times event *i* was immediately followed by event *j*. In this manner, we construct a set of log graphs $\{\mathcal {G}_{1},...,\mathcal {G}_{m},...,\mathcal {G}_{M}\}$.

We use these definitions to define graph-based log anomaly detection:

#### Problem 1 (Graph-based Log Anomaly Detection)

Given a set of attributed, directed, and weighted graphs that represent logs, find those graphs that are notably different from the majority of graphs.

What we mean by ‘notably different’ will have to be made more specific when we define our method, but we can already discuss what types of anomalies can potentially be detected. Most methods aim to detect two types of anomalies:

- A log group (here a graph) is considered a *quantitative anomaly* if the occurrence frequencies of some events in the group are higher or lower than expected from what is commonly observed. For example, if a file is opened (event *A*) twice, it should normally also be closed (event *B*) twice. In other words, the number of event occurrences $\#A = \#B$ in a normal pattern and an anomaly is detected if $\#A \ne \#B$.
- A log group is considered to contain *sequential anomalies* if the order of certain events violates the normal order pattern. For instance, a file can be closed only after it has been opened in a normal workflow. In other words, the order of event occurrences *A → B* is considered normal while *B → A* is considered anomalous.

An advantage of graph-based anomaly detection is that it can detect these two types of anomalies, but also anomalies reflected in the structures of the graphs. Moreover, no *unsupervised* log anomaly detection approaches represent event logs as attributed, directed, weighted graphs, which allow for even higher expressiveness than undirected graphs (and thus limiting the information loss resulting from the representation of the log files as graphs).

## Preliminaries: digraph inception convolutional nets

To learn node representations for attributed, directed, and edge-weighted graphs, Tong et al. (2020a) proposed Digraph Inception Convolutional Networks (DiGCN).

Specifically, given a graph $\mathcal {G}$ described by an adjacency matrix ${\mathbf {A}} \in \mathbb {R}^{|\mathcal {V}|\times |\mathcal {V}|}$, a node attribute matrix ${\mathbf {X}} \in \mathbb {R}^{|\mathcal {V}|\times d}$, and an edge-weight matrix ${\mathbf {Y}} \in \mathbb {R}^{|\mathcal {V}|\times |\mathcal {V}|}$, DiGCN defines the *k*-th order digraph convolution as

1 $$ {\mathbf{Z}}^{{(k)}} = \left\{ {\begin{array}{*{20}l} {{\mathbf{X}}\Theta ^{{(0)}} } \hfill & {k{\text{ = 0}},} \hfill \\ {\Psi {\mathbf{X}}\Theta ^{{(1)}} } \hfill & {k{\text{ = 1}},} \hfill \\ {\Phi {\mathbf{X}}\Theta ^{{(k)}} } \hfill & {k \ge {\mathrm{2}},} \hfill \\ \end{array} } \right. $$

where * Ψ * = $$ \frac{1}{2} $$ ($$ \Pi ^{{(1)\frac{1}{2}}} {\mathbf{P}}^{{(1)}} $$$$ {\Pi ^{{(1)\frac{{ - 1}}{2}}} } $$ + $$ {\Pi ^{{(1)\frac{{ - 1}}{2}}} } $$$$ {{\mathbf{P}}^{{(1)T}} \Pi ^{{(1)\frac{1}{2}}} } $$) and * Φ * = $$ {\mathbf{W}}^{{(k)\frac{{ - 1}}{2}}} $$$$ {\mathbf{P}}^{{(k)}} {\mathbf{W}}^{{(k)\frac{{ - 1}}{2}}}.$$ Particularly, $${\mathbf {Z}}^{(k)} \in \mathcal {R}^{|\mathcal {V}|\times f }$$ denotes the convolved output with *f* output dimension, and $$\Theta ^{(0)},\Theta ^{(1)},\Theta ^{(k)}$$ represent the trainable parameter matrices.

Moreover, ${\mathbf {P}}^{(k)}$ is the *k*-th order proximity matrix defined as

2 $$\begin{aligned} {\mathbf{P}}^{{(k)}} = \left\{ {\begin{array}{*{20}l} {\mathbf{I}} & {{\text {k = 0}} ,} \\ {\widetilde{{\mathbf{D}}}^{{ - 1}} \widetilde{{\mathbf{A}}}} & {{\text {k = 1}},} \\ {Ins\left( {({\mathbf{P}}^{{(1)}} )^{{(k - 1)}} ({\mathbf{P}}^{{(1)T}} )^{{(k - 1)}} } \right) } & {{\text {k}} \ge {\text {2}},} \\ \end{array} } \right. \end{aligned}$$

where ${\mathbf {I}} \in \mathcal {R}^{|\mathcal {V}|\times |\mathcal {V}|}$ is an identity matrix, $\tilde{{\mathbf {A}}} = {\mathbf {A}} + {\mathbf {I}}$, and $\tilde{{\mathbf {D}}}$ denotes the diagonal degree matrix with $\tilde{{\mathbf {D}}}_{ii} = \sum _{j}\tilde{{\mathbf {A}}}_{ij}$. Besides, $Ins\left( ({\mathbf {P}}^{(1)})^{(k-1)}({\mathbf {P}}^{(1)T})^{(k-1)}\right)$ is defined as

$$\begin{aligned} \frac{1}{2}Intersect\left( ({\mathbf {P}}^{(1)})^{(k-1)}({\mathbf {P}}^{(1)T})^{(k-1)},({\mathbf {P}}^{(1)T})^{(k-1)}({\mathbf {P}}^{(1)})^{(k-1)}\right) , \end{aligned}$$

with *Intersect(· )* denoting the element-wise intersection of two matrices (see Tong et al. (2020a) for computation details). In addition, ${\mathbf {W}}^{(k)}$ is the diagonalized weight matrix of ${\mathbf {P}}^{(k)}$, and $\Pi ^{(1)}$ is the approximate diagonalized eigenvector of ${\mathbf {P}}^{(1)}$. Particularly, the approximate diagonalized eigenvector is calculated based on personalized PageRank (Bahmani et al. 2010), with a parameter *α * to control the degree of conversion from a digraph to an undirected graph. We omit the details to conserve space, and refer to Tong et al. (2020a) for more details.

**Fig. 1:**
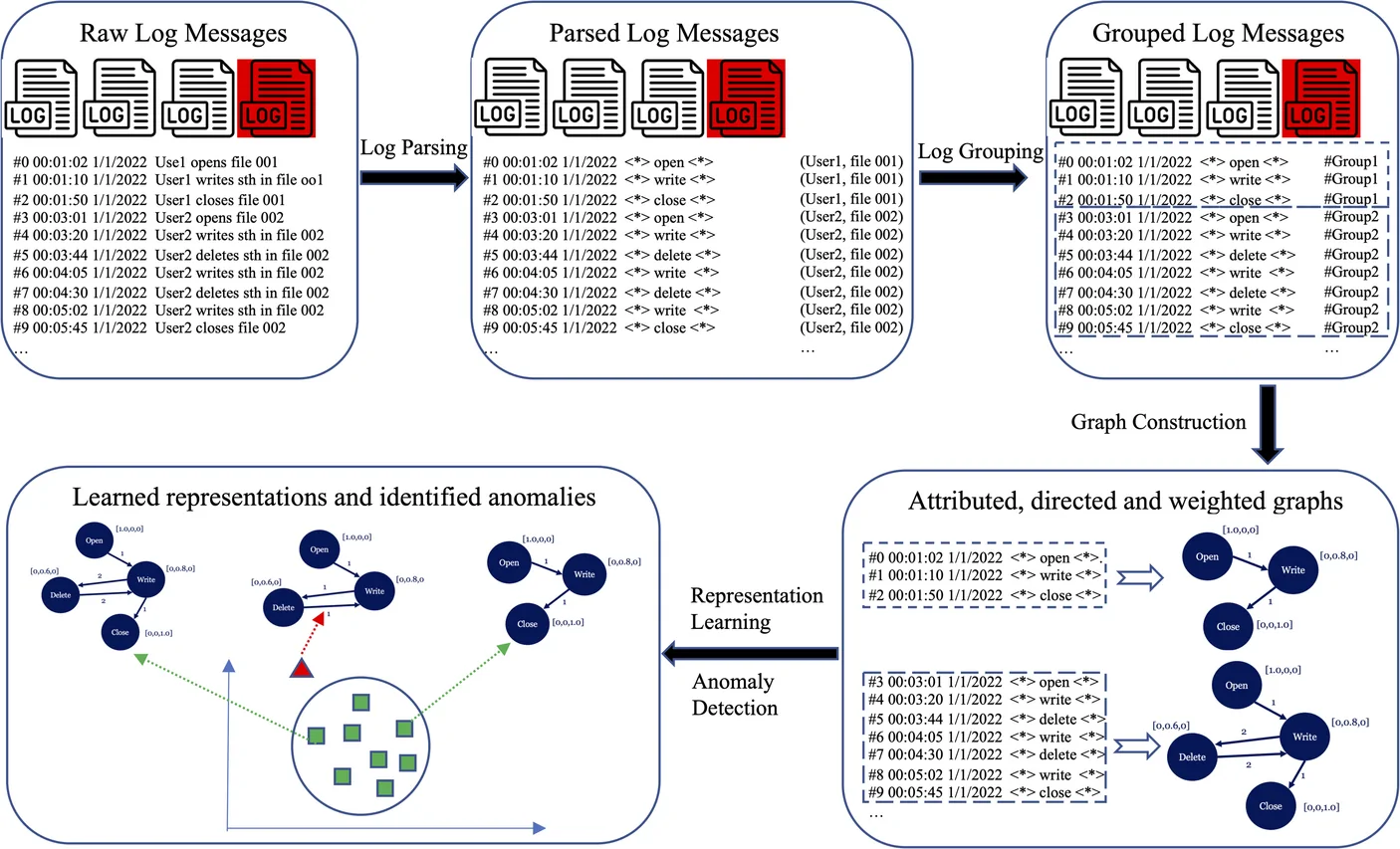
The Logs2Graphs pipeline. We use attributed, directed, and weighted graphs for representing the log files with high expressiveness, and integrate representation learning and anomaly detection for accurate anomaly detection. We use off-the-shelf methods for log parsing, log grouping, and graph construction

After obtaining the multi-scale features $\{{\mathbf {Z}}^{(0)},{\mathbf {Z}}^{(1)},...,{\mathbf {Z}}^{(k)}\}$, DiGCN defines an Inception block as

3 $$\begin{aligned} {\mathbf {Z}} = \sigma \left( \Gamma \left( {\mathbf {Z}}^{(0)},{\mathbf {Z}}^{(1)},...,{\mathbf {Z}}^{(k)}\right) \right) , \end{aligned}$$

where *σ * represents an activation function, and $\Gamma (\cdot )$ denotes a fusion operation, which can be summation, normalisation, and concatenation. In practice, we often adapt a fusion operation that keeps the output dimension unchanged, namely ${\mathbf {Z}} \in \mathcal {R}^{|\mathcal {V}|\times f}$. As a result, the *i*-th row of ${\mathbf {Z}}$ (namely ${\mathbf {Z}}_{i}$) denotes the learned vector representation for node $v_{i}$ in a certain layer.

## Graph-based anomaly detection for event logs

We propose *Logs2Graphs*, a graph-based log anomaly detection method tailored to event logs. The overall pipeline consists of the usual main steps, i.e., log parsing, log grouping, graph construction, graph representation learning, and anomaly detection, and is illustrated in Fig. 1. Note that we couple the graph representation learning and anomaly detection steps to accomplish end-to-end learning once the graphs have constructed.

First, after collecting logs from a system, the *log parsing* step extracts log events and log parameters from raw log messages. Since log parsing is not the primary focus of this article, we use Drain (He et al. 2017) for this task. Drain is a log parsing technique with fixed depth tree, and has been shown to generally outperform its competitors (Zhu et al. 2019). We make the following assumptions on the log files:

- Logs files are written in English;
- Each log message contains at least the following information: date, time, operation detail, and log identifier;
- The logs contain enough events to make the mined relationships (quantitative, sequential, structural) statistically meaningful, i.e., it must be possible to learn from the logs what the ‘normal’ behaviour of the system is.

Second, the *log grouping* step uses the log identifiers to divide the parsed log messages into log groups. Third, for each resulting group of log messages, the *graph construction* steps builds an attributed, directed, and edge-weighted graph, as described in more detail in Sect. 5.1. Fourth and last, in an integrated step for *graph representation learning and anomaly detection*, we learn a One-Class Digraph Inception Convolutional Network (OCDiGCN) based on the obtained set of log graphs. The resulting model can be used for graph-level anomaly detection. This model couples the graph representation learning objective and anomaly detection objective, and is thus trained in an end-to-end manner. The model, its training, and its use for graph-level anomaly detection are explained in detail in Sect. 5.2.

### Graph construction

**Fig. 2:**
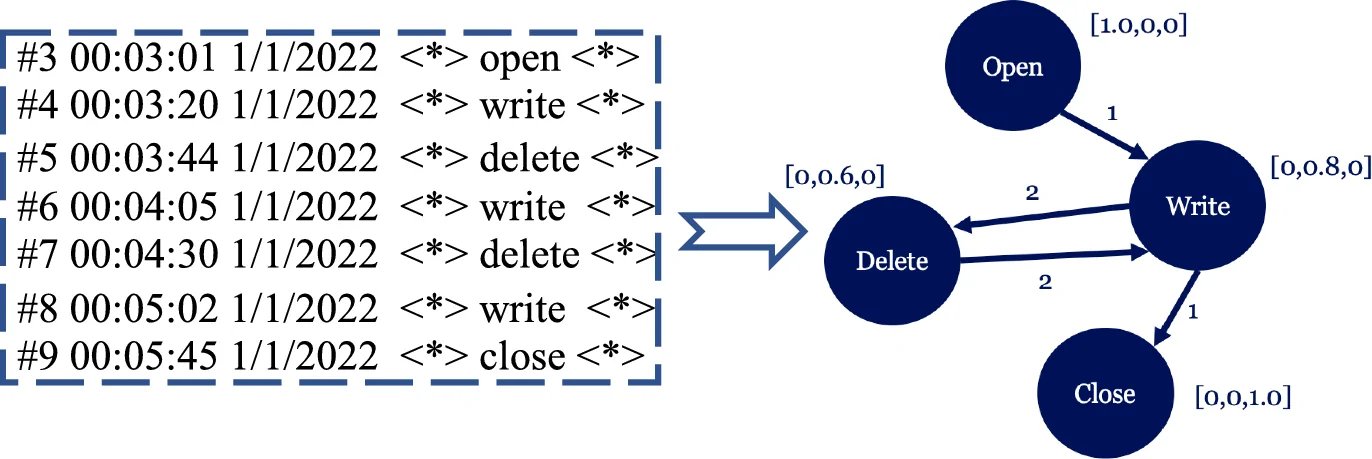
The construction of an attributed, directed, and edge-weighted graph from a group of log messages

We next explain how to construct an attributed, directed, and edge-weighted graph given a group of parsed log messages, and illustrate this in Fig. 2.

First, we utilise nodes to represent different log events. As a result, the number of nodes depends on the number of unique log events that occur within the log group. Second, starting from the first line of log messages in chronological order, we add a directed edge from log event $L_{i}$ to $L_{j}$ and set its edge-weight to 1 if the next event after $L_{i}$ is $L_{j}$. If the corresponding edge already exists, we increase its edge-weight by 1. In this manner, we obtain a labelled, directed, and edge-weighted graph.

However, using only the labels (e.g., *open* or *write*) of log events for graph construction may lead to missing important information. That is, we can improve on this by explicitly taking the semantic information of log events into account, by which we mean that we should look at the text of the log event in entirety. Specifically, we generate a vector representation for each log event as follows:

1. *Preprocessing*: for each log event, we first remove non-character words and stop words, and split compound words into separate words;
2. *Word embedding*: we use Glove (Pennington et al. 2014), a pre-trained word embedding model with 200 embedding dimensions to generate a vector representation for each word in a log event;
3. *Sentence embedding*: we generate a vector representation for each log event. Since the words in a sentence are usually not of equal importance, we use Term Frequency-Inverse Document frequency (TF-IDF) (Ramos 2003) to measure the importance of words. As a result, the weighted sum of word embedding vectors composes the vector representation of a log event.

By augmenting the nodes with the vector representations of the log events as attributes, we obtain an attributed, directed, and edge-weighted graph.

#### Discussion

The proposed graph construction is designed to capture key structural and semantic information from log data in a simple and effective manner. At the same time, several aspects can be further extended in future work.

(i) *Temporal information.* While the construction preserves the temporal ordering of log events within a log group via directed edges and captures interaction strength through edge weights, it does not explicitly encode absolute timestamps, inter-event time gaps, or event durations as node or edge attributes. Incorporating richer temporal information, such as duration or time-gap features, or adopting temporal or dynamic graph representations, constitutes a promising direction for future work.
(ii) *Log grouping strategy.* The current construction aggregates log events within a predefined log group, which determines the granularity at which normal behavior is learned. Although such grouping strategies (e.g., identifier-based or window-based grouping) are commonly adopted in log anomaly detection, different log groups may naturally exhibit varying activity levels during normal operation. More adaptive grouping strategies that account for heterogeneous or evolving logging patterns may further improve robustness in practice.
(iii) *Log type relationships.* In the current formulation, log events are treated as distinct nodes without explicitly encoding semantic relationships among different log types (e.g., warnings, errors, and informational messages). While such relationships may be implicitly reflected through co-occurrence patterns and temporal dependencies in the constructed graph, explicitly modeling log-type semantics or causal relations could further improve interpretability and detection capability.

### OCDiGCN: one-class digraph inception convolutional nets

We next describe One-Class Digraph Inception Convolutional Networks, abbreviated as OCDiGCN, a novel method for end-to-end graph-level anomaly detection. We chose to build on Digraph Inception Convolutional Networks (DiGCN) (Tong et al. 2020a) for their capability to handle directed graphs, which we argued previously is an advantage in graph-based log anomaly detection.

Given that DiGCN was designed for node representation learning, we repurpose it for graph representation learning as follows:

4 $$\begin{aligned} {\mathbf {z}} = \textrm{Readout}({\mathbf {Z}}_{i} \mid i\in \{1,2,...,|\mathcal {V}|\}). \end{aligned}$$

That is, at the final iteration layer, we utilise a so-called $\textrm{Readout}(\cdot )$ function to aggregate node vector representations to obtain a graph vector representation. Importantly, $\textrm{Readout}(\cdot )$ can be a simple permutation-invariant function such as maximum, sum or mean, or a more advanced graph-level pooling function (Ying et al. 2018).

Next, note that DiGCN work did not explicitly enable learning edge features (i.e., ${\mathbf {Y}}$). However, as DiGCN follows the Message Passing Neural Network (MPNN) framework (Gilmer et al. 2017), incorporating ${\mathbf {Y}}$ into Eq. (1) and conducting computations in Eqs. (2–4) analogously enables learning edge features.

Now, given a set of graphs $\{\mathcal {G}_{1},...,\mathcal {G}_{m},...,\mathcal {G}_{M}\}$, we can use Eq. (4) to obtain an explicit vector representation for each graph, respectively. We denote the vector presentation of $\mathcal {G}_{m}$ learned by the DiGCN model as $\mathrm {DiGCN(\mathcal {G}_{m};\mathcal {H})}$.

In graph anomaly detection, anomalies are typically identified based on a reconstruction or distance loss (Kim et al. 2022). In particular, the One-Class Deep SVDD objective (Ruff et al. 2018) is commonly used for two reasons: it can be easily combined with other neural networks, and more importantly, it generally achieves a state-of-the-art performance (Pang et al. 2021). To detect anomalies, we thus train a one-class classifier by optimizing the following One-Class Deep SVDD objective:

5 $$\begin{aligned} \min \limits _{\mathcal {H}}\frac{1}{M}\sum _{m=1}^{M}\Vert \mathrm {DiGCN(\mathcal {G}_{m};\mathcal {H})-{\mathbf {o}}}\Vert _{2}^{2}+\frac{\lambda }{2}\sum _{l=1}^{L}\Vert {\mathbf {H}}^{(l)}\Vert _{F}^{2}, \end{aligned}$$

where ${\mathbf {H}}^{(l)}$ represents the trainable parameters of DiGCN at the *l*-th layer, namely $(\Theta ^{(0)(l)},\Theta ^{(1)(l)},...,\Theta ^{(k)(l)})^{T}$, $\mathcal {H}$ denotes $\{{\mathbf {H}}^{(1)},...,{\mathbf {H}}^{(L)}\}$, *λ >0* represents the weight-decay hyperparameter, $\Vert \cdot \Vert _{2}$ is the Euclidean norm, and $\Vert \cdot \Vert _{F}$ denotes the Frobenius norm. Moreover, ${\mathbf {o}}$ is the center of the hypersphere in the learned representation space. Ruff et al. (2018) empirically found that setting ${\mathbf {o}}$ to the average of the network representations (i.e., graph representations in our case) obtained by performing an initial forward pass is a good strategy.

Ruff et al. (2018) also pointed out, however, that One-Class Deep SVDD classification may suffer from a hypersphere collapse, which will yield trivial solutions, namely mapping all graphs to a fixed center in the representation space. To avoid a hypersphere collapse, the hypersphere center ${\mathbf {o}}$ is set to the average of the network representations, the bias terms in the neural networks are removed, and unbounded activation functions such as ReLU are preferred.

After training the model on a set of non-anomalous graphs (or with a very low proportion of anomalies), given a test graph $\mathcal {G}_{m}$, we define its distance to the center in the representation space as its anomaly score, namely

6 $$\begin{aligned} score(\mathcal {G}_{m}) = \Vert \mathrm {DiGCN(\mathcal {G}_{m};\mathcal {H})-{\mathbf {o}}}\Vert _{2}. \end{aligned}$$

**Fig. 3:**
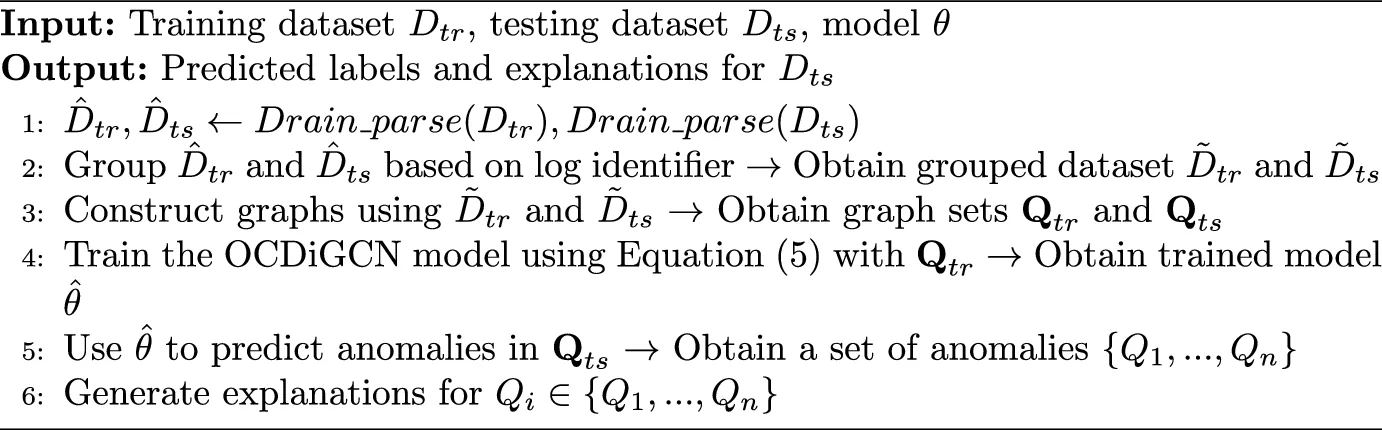
Pseudo-code of Logs2Graphs

**Training and hyperparameters: ** In summary, OCDiGCN is composed of an *L*-layer DiGCN architecture to learn node representations, plus a $\textrm{Readout}(\cdot )$ function to obtain the graph representation. It is trained in an end-to-end manner via optimising the SVDD objective, which can be optimised using stochastic optimisation techniques such as Adam (Kingma and Ba 2014). Overall, OCDiGCN takes a collection of non-anomalous graphs and a set of hyperparameters, which are outlined in Table 2, as inputs. The pseudo-code for Logs2Graphs is given in Algorithm 3.

**Choice of DiGCN.** We adopt DiGCN as the backbone of OCDiGCN primarily because it is designed to operate directly on directed graphs and provides a principled inception-based mechanism for capturing higher-order proximities. In log graphs, directed edges preserve temporal execution order between events, and anomalous behavior may manifest not only as unexpected immediate transitions, but also as deviations in longer-range execution patterns spanning multiple steps. DiGCN’s proximity-based formulation explicitly supports aggregating information from neighborhoods at different hop distances, which aligns well with this multi-scale nature of log-derived graphs. Although benchmark datasets typically yield relatively small graphs where first-order neighborhood aggregation (*k=1*) is sufficient (see Sect. 6.9), real-world logs may produce much larger graphs under identifier-based grouping. In such cases, higher-order proximity allows a node to incorporate information from more distant but semantically related events, enabling the model to capture abnormal execution flows that are not evident from immediate transitions alone. Recent independent work by Sarkima (2025) empirically confirms this behavior: on large industrial log graphs, increasing the proximity parameter to *k=2* significantly improves detection performance. This evidence highlights the practical relevance of DiGCN’s inception-based design, which offers both local sensitivity and the flexibility to model longer-range dependencies when required.

#### Alternative directed GNN backbones

DiGCN was not selected in isolation. Representative pre-2022 directed graph architectures form a clear line of development, from motif-based and directed-Laplacian reformulations such as MotifNet (Monti et al. 2018) and spectral-based GCNs for directed graphs (Ma et al. 2019), to more scalable directional propagation models such as DGCN (Tong et al. 2020b) and DiGCN (Tong et al. 2020a), and later magnetic-Laplacian approaches such as MagNet (Zhang et al. 2021). Within this line, we choose DiGCN because it operates directly on generic directed graphs, offers a principled proximity-based mechanism for multi-hop aggregation, and integrates naturally with our graph-level readout and one-class objective. At the same time, these models were introduced mainly for node classification or link prediction rather than *unsupervised graph-level anomaly detection* on attributed, directed, and edge-weighted log graphs. A fair benchmark would therefore require adapting each backbone to a common graph-level readout and Deep SVDD-style training pipeline, rather than simply swapping encoders. The present study does not attempt such a full directed-backbone benchmark under a unified protocol; we therefore regard it as valuable future work and list it explicitly as a limitation in Sect. 7.

#### Technical contribution of OCDiGCN

OCDiGCN does not claim a new generic graph convolution operator or a new one-class objective in isolation. Rather, its technical contribution lies in the *algorithmic adaptation* required to make directed graph convolution work coherently in a previously unsupported setting: *unsupervised graph-level anomaly detection on attributed, directed, and edge-weighted log graphs*. This is more than appending a Readout function and an SVDD loss to an existing encoder. DiGCN was originally proposed for directed *node* representation learning, whereas our setting requires graph-level representations, one-class optimization, preservation of directionality and edge weights from log-derived graphs, and explanations that remain faithful to the final anomaly score. Concretely, OCDiGCN contributes this task-specific integration in three ways. First, it lifts DiGCN from node representation learning to graph-level anomaly detection by introducing an appropriate graph readout while preserving the directed and edge-weighted structure of log graphs. Second, it couples the resulting graph representation with the Deep SVDD objective in an end-to-end manner, so that the encoder is trained specifically for one-class anomaly detection rather than for generic representation quality. Third, it aligns the explanation mechanism with this distance-based anomaly score, yielding anomaly explanations tied directly to the actual decision function. The contribution of OCDiGCN therefore lies in enabling a new methodological capability for log anomaly detection, rather than in claiming DiGCN or SVDD themselves as new primitives.

### Anomaly explanation

We provide a model-faithful explanation for anomalous log groups by combining representation-level counterfactual analysis (Guidotti 2024) with post-hoc feature attribution. The goal of our explanation is to support human understanding of anomalous behaviors by identifying influential log events and their semantic characteristics, rather than to perform causal inference or automated root-cause diagnosis.

Recall that a graph $\mathcal {G}_m$ is identified as anomalous if its graph-level representation deviates significantly from the learned hypersphere center (Eq. 6). The graph-level representation is obtained by applying an attributable Readout*(· )* function (e.g., sum or mean pooling) over node representations in the penultimate layer (Eq. 4). This design enables a principled decomposition of the anomaly score with respect to individual node representations.

#### Representation-level counterfactual explanation

For a given anomalous graph $\mathcal {G}_m$, we quantify the importance of node $v_j$ by measuring the change in anomaly score when its learned representation $\mathbf{Z}_j$ is excluded from the readout operation. Formally, the importance score of node $v_j$ is defined as

7 $$\begin{aligned} \textrm{Imp}(v_j) = \frac{\left| score(\mathcal {G}_m) - score(\mathcal {G}_m \setminus \{\mathbf{Z}_j\}) \right| }{score(\mathcal {G}_m)}, \end{aligned}$$

where $score(\mathcal {G}_m)$ denotes the anomaly score defined in Eq. 6, and $score(\mathcal {G}_m \setminus \{\mathbf{Z}_j\})$ is obtained by excluding $\mathbf{Z}_j$ when computing the graph-level representation.

This counterfactual analysis is performed at the representation level rather than directly perturbing the input graph. Directly removing nodes or edges at the input level would introduce cascading effects in message passing, making it difficult to attribute changes in the anomaly score to individual components. In contrast, representation-level counterfactuals provide a controlled and stable explanation that directly reflects the model’s decision mechanism.

By ranking nodes according to their importance scores, we identify a small subset of influential log events. If these nodes are connected in the original graph, the induced subgraph highlights the key event interactions associated with the anomaly, providing an intuitive structural view of abnormal behavior.

#### Input-level feature explanation

While representation-level counterfactual analysis identifies which log events are most influential for an anomaly, it does not directly explain which input features of these events contribute to their importance. To bridge this gap, we employ GraphLIME (Huang et al. 2022), a local, post-hoc, and model-agnostic explanation method, to provide feature-level interpretations for the most influential nodes.

Specifically, for each highly influential node, GraphLIME fits a local linear surrogate model in the node’s neighborhood to estimate the contribution of input features to the node’s learned representation. In our setting, this enables identification of which semantic features of a log event (e.g., components derived from its textual representation) are most relevant to the anomaly. GraphLIME complements the proposed counterfactual explanation by providing interpretable input-level insights while remaining agnostic to the internal architecture of the anomaly detection model.

#### Discussion of alternative explanation methods

Multiple post-hoc explanation methods have been proposed for interpreting neural network models, including gradient-based attribution techniques such as Layer-wise Relevance Propagation (LRP) (Bach et al. 2015). In the context of graph neural networks, gradient-based and relevance-propagation methods are typically used to attribute importance to node features or edges, and can provide useful local insights in certain settings. However, recent studies have shown that such post-hoc, gradient-based GNN explanation methods can be fundamentally unreliable. In particular, Li et al. (2024a) demonstrate that a wide range of popular GNN explainers—including gradient-based, decomposition-based, and perturbation-based methods—are highly susceptible to prediction-preserving adversarial perturbations: small, imperceptible changes to the input graph that do not alter the model’s prediction can nevertheless lead to drastically different explanations. This instability calls into question the trustworthiness of gradient-based relevance propagation when explanations are expected to faithfully reflect the underlying anomaly scoring mechanism. In this work, we therefore favor local surrogate-based explanations over gradient-based relevance propagation. This choice is motivated by the objective of maintaining a clear and faithful alignment between the explanation and the distance-based anomaly scoring function. GraphLIME offers a simple and interpretable feature attribution mechanism that does not rely on backpropagation through the model and can be naturally combined with the proposed representation-level counterfactual analysis. We also note that structure-oriented explanation methods such as GNNExplainer (Ying et al. 2019) are primarily designed to preserve class predictions in supervised classification settings. Adapting such methods to a distance-based one-class anomaly detection objective is non-trivial and may introduce additional optimization complexity. Instead, our approach explains structural contributions through counterfactual analysis at the representation level, which directly reflects changes in the anomaly score.

Overall, the proposed explanation framework combines representation-level counterfactual reasoning with input-level feature attribution, providing interpretable and model-faithful explanations while maintaining a clear scope and avoiding overclaiming causal interpretations.

## Experiments

We perform extensive experiments to answer the following questions:

1. **Detection accuracy:** How effective is *Logs2Graphs* at identifying log anomalies when compared to state-of-the-art methods?
2. **Directed vs. undirected graphs**: Is the directed log graph representation better than the undirected version for detecting log anomalies?
3. **Node Labels vs. Node Attributes**: How important is it to use semantic embeddings of log events as node attributes?
4. **Robustness analysis:** To what extent is Logs2Graphs robust to contamination of the training data?
5. **Ability to detect structural anomalies:** Can Logs2Graphs better capture structural anomalies and identify structurally equivalent normal instances than its contenders?
6. **Explainability Analysis:** How understandable are the anomaly explanations given by Logs2Graphs?
7. **Sensitivity analysis:** How do the values of the hyperparameters influence the detection accuracy?
8. **Runtime analysis:** What are the runtimes for the different methods?

### Experiment setup

#### Datasets

The five datasets that we use, summarized in Table 1, were chosen for three reasons: (1) they are commonly used for the evaluation of log anomaly detection methods; (2) they contain ground truth labels that can be used to calculate evaluation metrics; and (3) they include log identifiers that can be used for partitioning log messages into groups. For each group of log messages in a dataset, we label the group as anomalous if it contains at least one anomaly. More details are given as follows:

- HDFS (Xu et al. 2009) consists of Hadoop Distributed File System logs obtained by running 200 Amazon EC2 nodes. These logs contain *block_id*, which can be used to group log events into different groups. Moreover, these logs are manually labeled by Hadoop experts.
- Hadoop (Lin et al. 2016) was collected from a Hadoop cluster consisting of 46 cores over 5 machines. The *ContainerID* variable is used to divide log messages into different groups.
- BGL, Spirit, and Thunderbird contain system logs collected from the BlueGene/L (BGL), Spirit, and Thunderbird supercomputing systems located at Sandia National Labs, respectively. For those datasets, each log message was manually inspected by engineers and labelled as normal or anomalous. For BGL, we use all log messages, and group log messages based on the *Node* variable. For Spirit and Thunderbird, we only use the first 1 million and first 5 million log messages for evaluation, respectively. Furthermore, for these two datasets, the *User* is used as log identifier to group log messages. However, considering that an ordinary user may generate hundreds of thousands of logs, we regard every 100 consecutive logs of each user as a group. If the number of logs is less than 100, we also consider it as a group.

We note that identifier-based grouping is a common and convenient choice in the log anomaly detection literature (He et al. 2016; Lu et al. 2018; Zhang et al. 2020; Le and Zhang 2022). When such identifiers are unavailable, alternative grouping strategies such as fixed-size or sliding windows can be adopted, although the choice of grouping strategy may influence detection performance (Le and Zhang 2022).

**Table 1:** Summary of datasets

Name	#Events	#Graphs	#Anomalies	#Nodes	#Edges
HDFS	48	575,061	16,838	7	20
Hadoop	683	978	811	34	120
BGL	1848	69,251	31,374	10	30
Spirit	834	10,155	4,432	6	24
Thunderbird	1013	52,160	6,814	16	52

#Events refers to the number of log event templates obtained using log parser Drain (He et al. 2017). #Groups means the number of generated graphs. #Anomalies represents the number of anomalous graphs. #Nodes denotes the average number of nodes in generated graphs. #Edges indicates the average number of edges in the generated graphs

**Table 2:** Description of hyperparameters involved in OCDiGCN

Symbol	Meaning	Range
*Bs*	Batch size	{16, 32, 64, **128**, 256, 512, 1024, 1536, 2048, 2560}
*Op*	Optimisation method	Adam, **SGD**
*L*	Number of layers	$\{{\mathbf {1}},2,3,4,5\}$
*λ *	Weight decay parameter	$\{{\mathbf {0.0001}}, 0.001,0.01,0.1\}$
*η *	Learning rate	$\{0.0001,0.001,{\mathbf {0.01}}\}$
*k*	Proximity parameter	$\{{\mathbf {1}},2\}$
*α *	Teleport probability	$\{0.05, {\mathbf {0.1}}, 0.2\}$
*Γ *	Fusion operation if *k≥ 2*	Sum, concatenation
*Re*	Readout function	**Mean**, sum, max
*d*	Embedding dimension	$\{32, 64, {\mathbf {128}}, 256, 300\}$
*Ep*	Epochs for training	Range(100,1000,50)

**Range** indicates the values that we have tried on validation data, and boldfaced values are the values suggested to use in experiments. Particularly, for the embedding dimensions: 300 is suggested for BGL and 128 for others. For the batch sizes: 32 is suggested for HDFS and 128 for others. For the training epochs: 100 for BGL and Thunderbird, 200 for HDFS, 300 for Hadoop and 500 for Spirit are suggested

#### Baselines

To investigate the performance of *Logs*2*Graphs*, we compare it with the following seven log anomaly detection methods: Principal Component Analysis (PCA) (Xu et al. 2009), One-Class SVM (OCSVM) (Schölkopf et al. 2001), Isolation Forest (iForest) (Liu et al. 2008), HBOS (Goldstein and Dengel 2012), DeepLog (Du et al. 2017), LogAnomaly (Meng et al. 2019), and AutoEncoder (Farzad and Gulliver 2020), and one state-of-the-art graph level anomaly detection method: GLAM (Zhao et al. 2022).

We choose these methods as baselines because they are often regarded to be representatives of traditional machine learning-based (PCA, OCSVM, IForest, HBOS) and deep learning-based approaches (DeepLog, LogAnomaly and AutoEncoder), respectively. All methods are unsupervised or semi-supervised methods that do not require labeled anomalous samples for training the models.

##### Clarification on excluded graph-based log baselines

We note that several closely related graph-based log anomaly detection methods, including LogGD, GLAD-PAW, and GLAD, are highly relevant to our work but are not included in the quantitative benchmark for different reasons. LogGD (Xie et al. 2022) is a supervised method that requires substantial labeled normal and anomalous training data, whereas our study focuses on the unsupervised/one-class setting and trains on normal data only. Directly comparing a supervised detector trained with anomaly labels against unsupervised methods trained without such labels would therefore not constitute an apples-to-apples evaluation. GLAD-PAW (Wan et al. 2021) does not provide publicly available code, which makes faithful reproduction difficult. GLAD (Li et al. 2023), in turn, constructs undirected, weighted, and attributed heterogeneous dynamic log graphs and focuses on anomalous relation/edge detection rather than graph-level anomaly detection on directed log graphs. To assess the impact of this key design choice, we include a controlled directed-versus-undirected comparison using GLAM in the corresponding subsection below. There, the undirected variant consistently underperforms *Logs2Graphs* on all datasets except Hadoop, supporting the practical importance of preserving edge directionality in our setting.

#### Evaluation metrics

The Area Under Receiver Operating Characteristics Curve (ROC AUC) and the Area Under the Precision-Recall Curve (PRC AUC) are widely used to quantify the detection accuracy of anomaly detection (Aggarwal and Aggarwal 2017). This is mainly because they can provide a single value that summarizes the overall performance of the anomaly detection model across various thresholds. In contrast, other metrics such as Precision, Recall and F1-score depend on choosing a threshold to determine whether an instance is anomalous or normal. Consequently, different thresholds can result in different values. Therefore, we employ ROC AUC and PRC AUC to evaluate and compare the different log anomaly detection methods. PRC AUC is also known as Average Precision (AP). For both ROC AUC and PRC AUC, values closer to 1 indicate better performance.

### Model implementation and configuration

Traditional machine learning based approaches—such as PCA, OCSVM, iForest, and HBOS—usually first transform logs into log event count vectors, and then apply traditional anomaly detection techniques to identify anomalies. For these methods, we utilise their open-source implementations provided in PyOD (Zhao et al. 2019). Meanwhile, for deep learning methods DeepLog, LogAnomaly, and AutoEncoder, we use their open-source implementations in Deep-Loglizer (Chen et al. 2021). For these methods, we use their default hyperparameter values.

For all deep learning based methods, the experimental design adopted in this study follows a train/validation/test strategy with a distribution of $70\%:5\%:25\%$ for normal instances. Specifically, the model was trained using *70%* of normal instances, while *5%* of normal instances and an equal number of abnormal instances were employed for validation (i.e., hyperparameter tuning). The remaining *25%* of normal instances and the remaining abnormal instances were used for testing. Table 2 summarizes the hyperparameters involved in OCDiGCN as well as their recommended values.

We implemented and ran all algorithms in Python 3.8 (using PyTorch (Paszke et al. 2019) and PyTorch Geometric (Fey and Lenssen 2019) libraries when applicable), on a workstation equipped with an Intel i7-11700KF CPU and Nvidia RTX3070 GPU. For reproducibility, all code and datasets are released on GitHub.^1^

**Table 3:** Anomaly detection accuracy on five benchmark datasets for *Logs2Graphs* and its eight competitors

	HDFS		Hadoop		BGL		Spirit		Thunderbird	
Method	AP	RC	AP	RC	AP	RC	AP	RC	AP	RC
PCA	*0.91* ± 0.03	**1**.**0** ± 0.00	0.84 ± 0.00	0.52 ± 0.00	0.73 ± 0.01	0.82 ± 0.00	0.31 ± 0.00	0.19 ± 0.00	0.11 ± 0.00	0.34 ± 0.01
OCSVM	0.18 ± 0.01	0.88 ± 0.01	0.83 ± 0.00	0.45 ± 0.00	0.47 ± 0.00	0.47 ± 0.01	0.34 ± 0.00	0.29 ± 0.00	0.12 ± 0.00	0.45 ± 0.01
IForest	0.73 ± 0.04	0.97 ± 0.01	0.85 ± 0.01	0.55 ± 0.01	0.79 ± 0.01	0.83 ± 0.01	0.32 ± 0.03	0.23 ± 0.02	0.11 ± 0.01	0.24 ± 0.10
HBOS	0.74 ± 0.04	*0.99* ± 0.00	0.84 ± 0.00	0.50 ± 0.00	0.84 ± 0.02	0.87 ± 0.03	0.35 ± 0.00	0.22 ± 0.00	0.15 ± 0.01	0.29 ± 0.05
DeepLog	**0**.**92** ± 0.07	0.97 ± 0.04	**0**.**96** ± 0.00	0.47 ± 0.00	0.89 ± 0.00	0.72 ± 0.00	*0.99* ± 0.00	*0.97* ± 0.00	*0.91* ± 0.01	*0.96* ± 0.00
LogAnomaly	0.89 ± 0.09	0.95 ± 0.05	**0**.**96** ± 0.00	0.47 ± 0.00	0.89 ± 0.00	0.72 ± 0.00	*0.99* ± 0.00	*0.97* ± 0.00	0.90 ± 0.01	*0.96* ± 0.00
AutoEncoder	0.71 ± 0.03	0.84 ± 0.01	**0**.**96** ± 0.00	0.52 ± 0.00	0.91 ± 0.01	0.79 ± 0.02	0.96 ± 0.00	0.92 ± 0.01	0.44 ± 0.02	0.46 ± 0.05
GLAM	0.78 ± 0.08	0.89 ± 0.04	*0.95* ± 0.00	**0**.**61** ± 0.00	*0.94* ± 0.02	*0.90* ± 0.03	0.93 ± 0.00	0.91 ± 0.00	0.75 ± 0.02	0.85 ± 0.01
Logs2Graphs	0.87 ± 0.04	0.91 ± 0.02	*0.95* ± 0.00	*0.59* ± 0.00	**0**.**96** ± 0.01	**0**.**93** ± 0.01	**1**.**0** ± 0.00	**1**.**0** ± 0.00	**0**.**99** ± 0.00	**1**.**0** ± 0.00

AP and RC denote Average Precision and ROC AUC, respectively. HDFS, BGL, and Thunderbird have been downsampled to 10,000 graphs each while maintaining the original anomaly rates. For each method on each dataset, to mitigate potential biases arising from randomness, we conducted ten experimental runs with varying random seeds and report the average values along with standard deviations of AP and RC. Moreover, we highlight the best results with **bold** and the runner-up with *Italic*

### Comparison to the state of the art

We first compare *Logs*2*Graphs* to the state of the art (as of the writing of this paper, namely the year of 2024). We have the following main observations according to the results in Table 3:

- In terms of ROC AUC, *Logs2Graphs* achieves the best performance against its competitors on three out of five datasets. Particularly, *Logs2Graphs* outperforms the closet competitor on BGL with 9.6% and delivers remarkable results (i.e., an ROC AUC larger than 0.99) on Spirit and Thunderbird. Similar observations can be made for Average Precision.
- Deep learning based methods generally outperform the traditional machine learning based methods. One possible reason is that traditional machine learning based methods only leverage log event count vectors as input, which makes them unable to capture and exploit sequential relationships between log events and the semantics of the log templates.
- The performance of (not-graph-based) deep learning methods is often inferior to that of *Log2Graphs* on the more complex datasets, i.e., BGL, Spirit, and Thunderbird, which all contain hundreds or even thousands of log templates. This suggests that LSTM-based models may not be well suited for logs with a large number of log templates. One possible reason is that the test dataset contains many unprecedented log templates, namely log templates that are not present in the training dataset.
- In terms of ROC AUC score, all methods except for OCSVM and AutoEncoder achieve impressive results (with *RC> 0.91*) on HDFS. One possible reason is that HDFS is a relatively simple log dataset that contains only 48 log templates. Concerning AP, PCA and LSTM-based DeepLog achieve impressive results (with *AP>0.89*) on HDFS. Meanwhile, *Logs2Graphs* obtains a competitive performance (with *AP=0.87*) on HDFS.

### Directed vs. undirected graphs

To investigate the practical added value of using *directed* log graphs as opposed to *undirected* log graphs, we convert the logs to attributed, undirected, and edge-weighted graphs, and apply GLAM (Zhao et al. 2022), a graph-level anomaly detection method for undirected graphs. We use the same graph construction method as for *Logs2Graphs*, except that we use undirected edges. Similar to our method, GLAM also couples the graph representation learning and anomaly detection objectives by optimising a single SVDD objective. The key difference with OCDiGCN is that GLAM leverages GIN (Xu et al. 2018), which can only tackle undirected graphs, while OCDiGCN utilizes DiGCN (Tong et al. 2020a) that is especially designed for directed graphs.

The results in Table 3 indicate that GLAM’s detection performance is comparable to that of most competitors. However, it consistently underperforms on all datasets, except for Hadoop, when compared to *Logs2Graphs*. Given that the directed vs undirected representation of the log graphs is the key difference between the methods, a plausible explanation is that directed graphs have the capability to retain the temporal sequencing of log events, whereas undirected graphs lack this ability. Consequently, GLAM may encounter difficulties in detecting sequential anomalies and is outperformed by *Logs2Graphs*.

### Node labels vs. node attributes

**Fig. 4:**
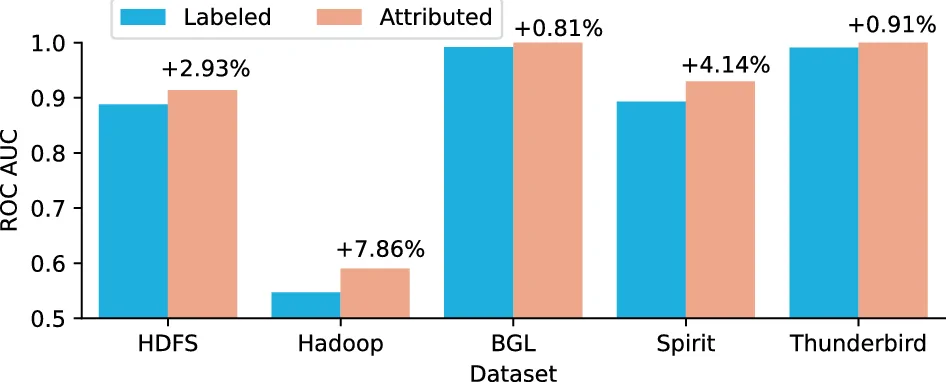
The comparative performance analysis of Logs2Graphs, measured by ROC AUC, demonstrating the distinction between utilizing node semantic attributes and node labels

To investigate the importance of using semantic embeddings of log events as node attributes, we replace the node semantic attributes with one-hot-encoding of node labels (i.e., using an integer to represent a log event). The performance comparisons in terms of ROC AUC for Logs2Graphs are depicted in Fig. 4, which shows that using semantic embeddings is always superior to using node labels. Particularly, it can lead to a substantial performance improvement on the Hadoop, Spirit and HDFS datasets. The PRC AUC results show a similar behaviour and thus are omitted.

### Robustness to contamination

**Fig. 5:**
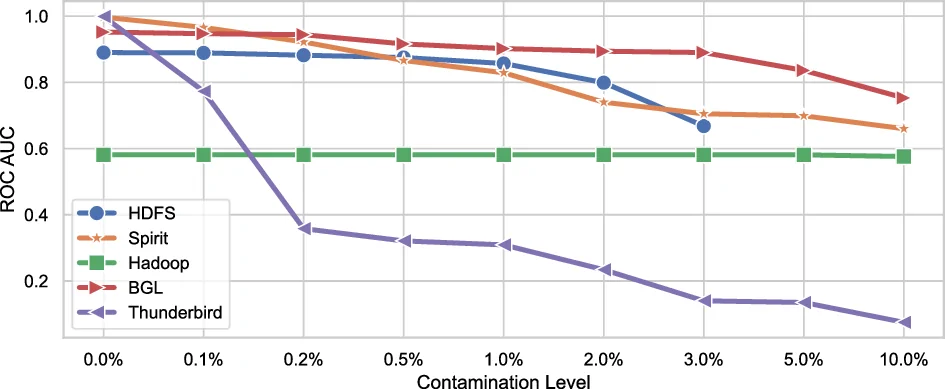
ROC AUC results of Logs2Graphs w.r.t. a wide range of contamination levels. Results are averaged over 10 runs. Particularly, HDFS contains only 3% anomalies and thus results at 5% and 10% are not available

To investigate the robustness of Logs2Graphs when the training dataset is contaminated (namely integrating anomalous graphs in training data), we report its performance in terms of ROC AUC under a wide range of contamination levels. Figure 5 shows that the performance of Logs2Graphs decreases with an increase of contamination in the training data. The PRC AUC results show a similar behaviour and thus are omitted. Hence, it is important to ensure that the training data contains only normal graphs (or with a very low proportion of anomalies).

**Practical considerations for training data selection.** While the above experiment highlights the sensitivity of Logs2Graphs to contaminated training data, this behavior is shared by many deep anomaly detection methods (Wu et al. 2022). In practice, high-quality normal training data can often be obtained from real systems by leveraging logs collected during controlled tests, stable operational periods, or successful executions, which are commonly treated as representative of normal behavior in industrial settings (Nguyen et al. 2019). When applying Logs2Graphs to new datasets, we recommend the following practical guidelines. First, training should start from log data that are known, or strongly believed, to correspond to normal system behavior. Second, robustness can be assessed by conducting contamination analysis or monitoring validation curves as small amounts of anomalous data are introduced. Third, as system behavior evolves over time, the model can be periodically retrained or updated using newly collected normal data. Recent independent evaluation on large-scale industrial logs further supports the practicality of this approach. In particular, Sarkima (2025) demonstrates that Logs2Graphs achieves strong detection performance on substantially larger and more complex telecommunications log graphs when trained on carefully selected normal data, even under realistic operational conditions.

### Ability to detect structural anomalies and recognise unseen normal instances

**Fig. 6:**
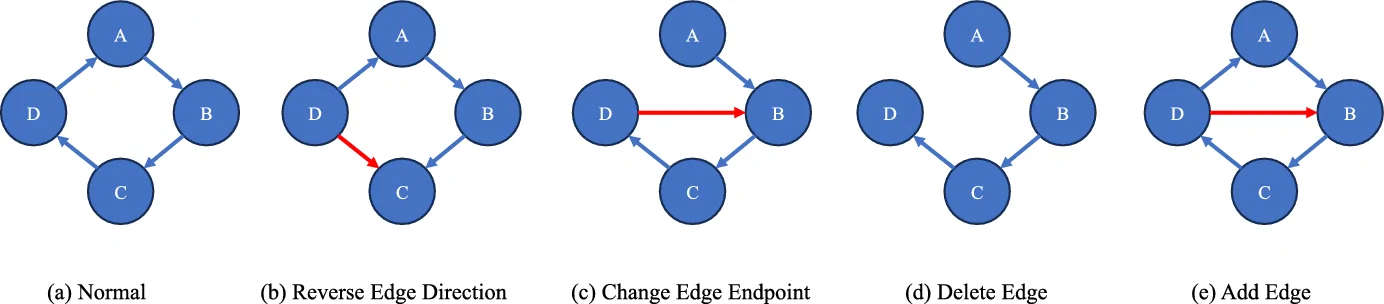
Synthetic generation of normal (10000) and structurally anomalous (200 each) graphs

To showcase the effectiveness of different neural networks in detecting structural anomalies, we synthetically generate normal and anomalous directed graphs as shown in Fig. 6. As Deeplog, LogAnomaly and AutoEncoder require log sequences as inputs, we convert directed graphs into sequences by sequentially presenting the endpoints pair of each edge. Moreover, for GLAM we convert directed graphs into undirected graphs by turning each directed edge into an undirected edge.

Moreover, to investigate their capability of recognising unseen but structurally equivalent normal instances, we generate the following normal log sequences based on the synthetic normal graph as training data: $A \rightarrow B \rightarrow C \rightarrow D \rightarrow A$ (1000), $B \rightarrow C \rightarrow D \rightarrow A \rightarrow B$ (1000) and $C \rightarrow D \rightarrow A \rightarrow B \rightarrow C$ (1000), and the following as test dataset: $D \rightarrow A \rightarrow B \rightarrow C \rightarrow D$ (1000).

**Table 4:** ROC AUC results (higher is better) of detecting structural anomalies and False Positive Rate (lower is better) of recognising unseen normal instances

Case	Deeplog	LogAnomaly	AutoEncoder	GLAM	Ours
S1 (ROC)	1.0	1.0	0.0	0.0	1.0
S2 (ROC)	1.0	1.0	0.50	1.0	1.0
S3 (ROC)	1.0	1.0	1.0	1.0	1.0
S4 (ROC)	1.0	1.0	1.0	1.0	1.0
N1 (FPR)	100%	100%	100%	0%	0%

S1: Reverse Edge Direction; S2: Change Edge Endpoint; S3: Delete Edge; S4: Add Edge; N1: Unseen normal instances

The results in Table 4 indicate that Logs2Graphs, Deeplog and LogAnomaly can effectively detect structural anomalies, while AutoEncoder and GLAM fail in some cases. However, log sequences based methods, namely Deeplog, LogAnomaly and AutoEncoder, can lead to high false positive rates due to their inability of recognising unseen but structurally equivalent normal instances.

**Fig. 7:**
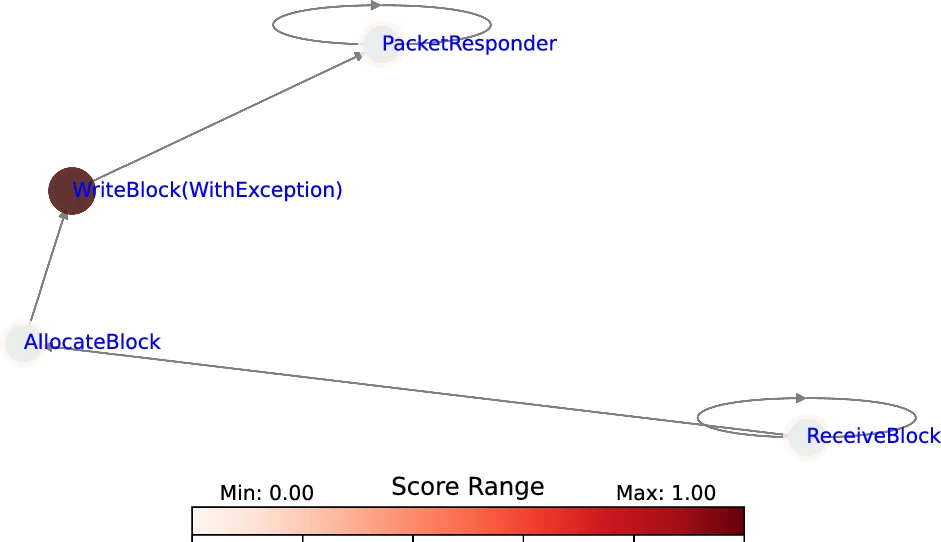
Example of anomaly explanation with HDFS (the log event templates are simplified for better visualisation)

### Anomaly explanation

Fig. 7 provides an example of log anomaly explanation with the HDFS dataset. For each detected anomalous log graph (namely a group of logs), we first quantify the importance of nodes according to the description in Sect. 5.3. Next, we visualise the anomalous graph by assigning darker shade of red to more important nodes. In this example, the node “WriteBlock(WithException)” contributes the most to the anomaly score of an anomalous log group and thus is highlighted in red.

### Sensitivity analysis

We examine the effects of four hyperparameters in OCDiGCN on the detection performance, including the number of convolutional layers *L*, the embedding dimension *d*, the proximity parameter *k*, and the teleport probability *α *.

**Fig. 8:**
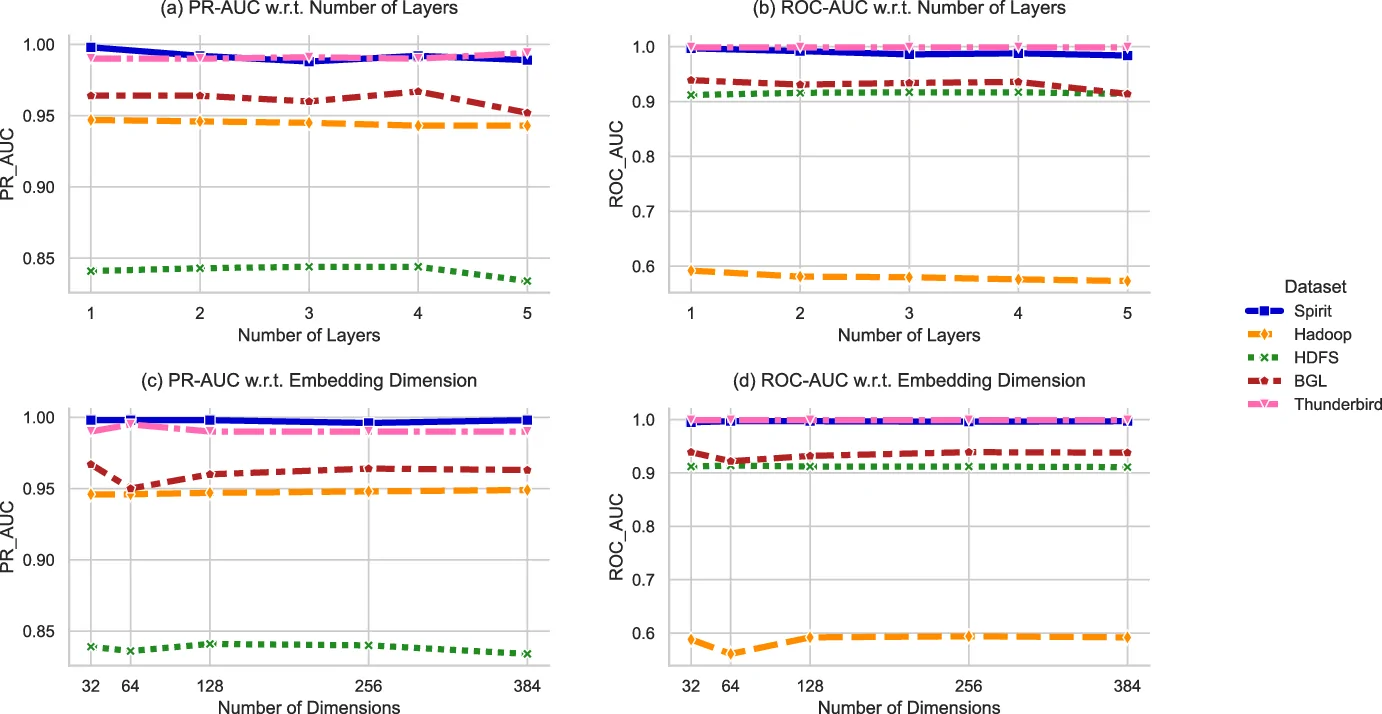
The effects of the number of layers (top row) and the embedding dimensions (bottom row) on AP (left column) and ROC AUC (right column)

**The Number of Convolutional Layers:** *L* is a potentially important parameter as it determines how many convolutional layers to use in OCDiGCN. Figure 8 (top row) depicts PRC AUC and ROC AUC for the five benchmark datasets when *L* is varied from 1 to 5. We found that *L=1* yields consistently good performance. As the value of *L* is increased, there is only a slight enhancement in the resulting performance or even degradation, while the associated computational burden increases substantially. We thus recommend to set *L=1*.

**The Embedding Dimension** *d* **:** From Figure 8 (bottom row), one can see that *d=128* yields good performance on Spirit, Hadoop, HDFS and Thunderbird, while further increasing *d* obtains negligible performance improvement or even degradation. However, an increase of *d* on BGL leads to substantially better performance. One possible reason is that BGL is a complex dataset wherein anomalies and normal instances are not easily separable on lower dimensions.

**Fig. 9:**
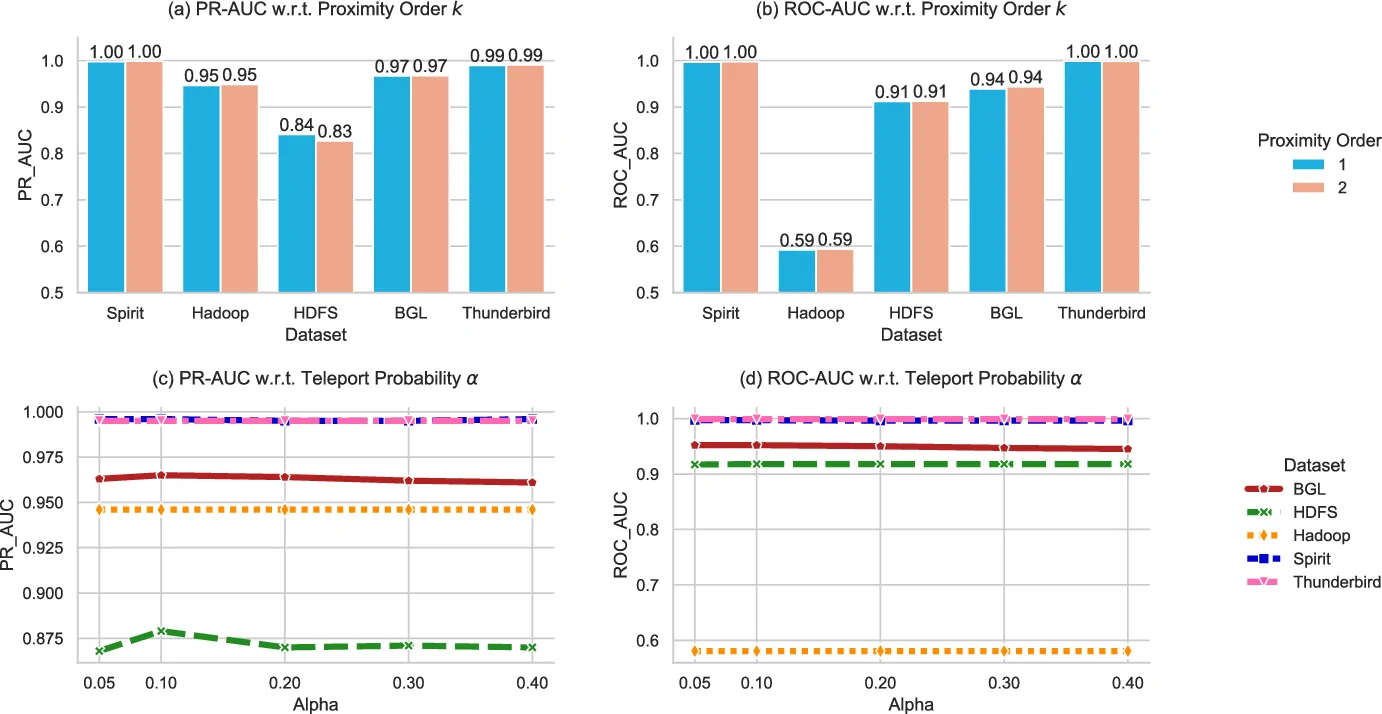
The effects of the proximity parameter *k* (top row) and the teleport probability *α * (bottom row) on AP (left column) and ROC AUC (right column)

**The Proximity Parameter** *k* **:** As this parameter increases, a node can gain more information from its further neighbours. Figure 9 (top row) contrasts the detection performance when *k* is set to 1 and 2, respectively. Particularly, we construct one Inception Block when *k = 2*, using concatenation to fuse the results. We observe that there is no large improvement in performance when using a value of *k=2* in comparison to *k=1*. It is important to recognize that a node exhibits 0th-order proximity with itself and 1st-order proximity with its immediately connected neighbors. If *k=2*, a node can directly aggregate information from its 2nd-order neighbours. As described in Table 1, graphs generated from logs usually contain a limited number of nodes, varying from 6 to 34. Therefore, there is no need to utilise the Inception Block, which was originally designed to handle large graphs in Tong et al. (2020a).

**The Teleport Probability** *α *: The teleport probability *α * is a key hyperparameter in DiGCN that controls the strength of the digraph-to-undigraph conversion. To analyze its impact, we evaluate OCDiGCN under a wider range of values $\alpha \in \{0.05, 0.1, 0.2, 0.3, 0.4\}$. Figure 9 (bottom row) reports the corresponding PR and ROC AUC results. Overall, the detection performance remains relatively stable across this range, indicating that OCDiGCN is not sensitive to the choice of *α *. In particular, *α * = 0.1 consistently achieves strong or near-optimal performance across datasets, which supports our default setting used in the experiments. These results suggest that *α * mainly acts as a stabilizing parameter rather than a highly sensitive tuning factor in log anomaly detection.

### Runtime analysis

Traditional machine learning methods, including PCA, OCSVM, IForest and HBOS, usually perform log anomaly detection in a transductive way. In other words, they require the complete dataset beforehand and do not follow a train-and-test strategy. In contrast, neural network based methods, such as DeepLog, LogAnomaly, AutoEncoder, and Logs2Graphs, perform log anomaly detection in an inductive manner, namely following a train-and-test strategy.

**Fig. 10:**
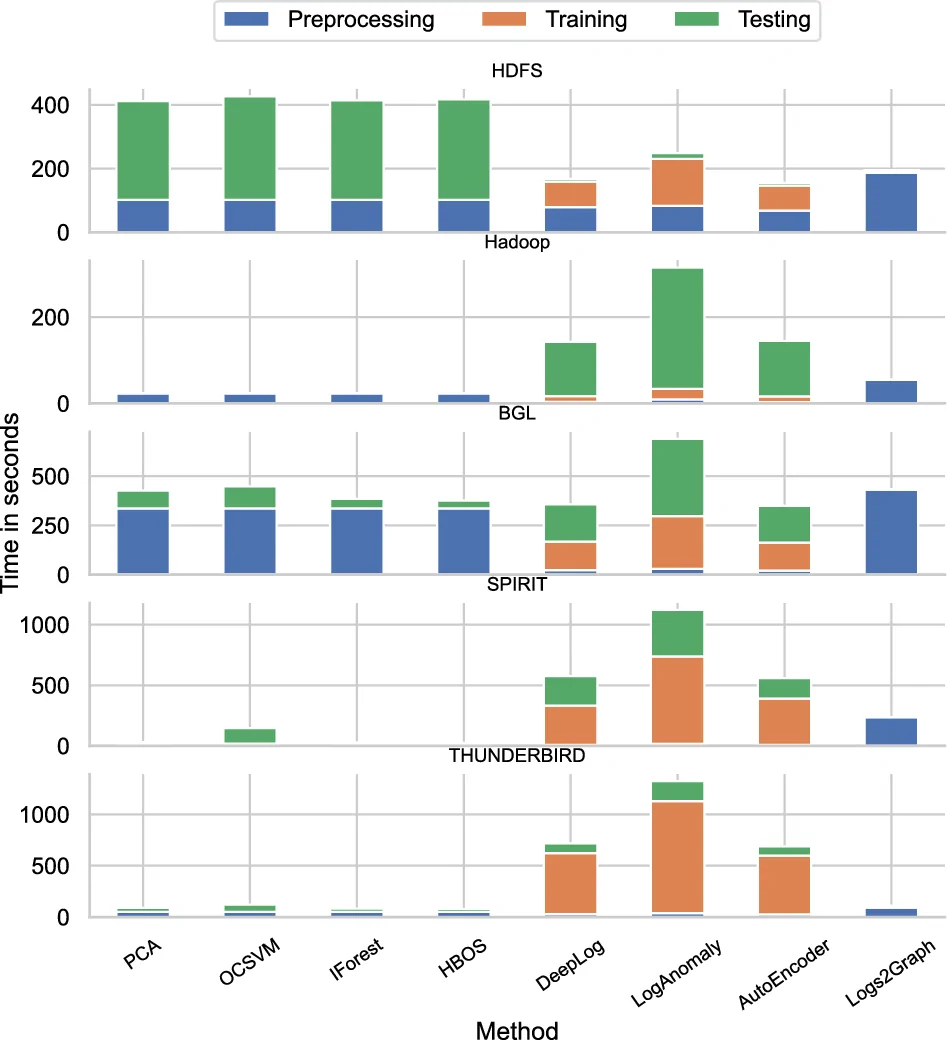
Runtime for all eight methods on all datasets, wherein HDFS, BGL, and Thunderbird have been downsampled to 10,000 graphs. Runetimes are averaged over 10 repetitions. We report the training time per epoch for neural network based methods

Figure 10 shows that most computational time demanded by *Logs2Graphs* is allocated towards the graph generation phase. In contrast, the training and testing phases require a minimal time budget. The graph generation phase can be amenable to parallelisation though, thereby potentially reducing the overall processing time. As a result, *Logs2Graphs* shows great promise in performing online log anomaly detection. Meanwhile, other neural networks based models—such as DeepLog, LogAnomaly, and AutoEncoder—demand considerably more time for the training and testing phases.

Scalability considerations. We analyze the computational and memory characteristics of *Logs2Graphs* with respect to the number of log messages per group and the resulting graph size. Let a log group contain *n* parsed log messages and |*L*| unique log events (templates). The constructed graph has *|V|=|L|* nodes and at most *|E|≤ n-1* directed edges (before merging repeated transitions by edge weights). Graph construction can be implemented in a single pass over the *n* messages: mapping each message to a template ID and updating an edge-weight counter for each consecutive pair. Using a hash map (or sparse accumulator) for edge counting, this yields time complexity $\mathcal {O}(n)$ and memory complexity $\mathcal {O}(|V|+|E|)$ for each group.

**Effect of |** *L* **|, |** *V* **|, and |** *E* **|.** The downstream GNN computation scales with the graph size per log group rather than the total log volume. In our implementation, OCDiGCN is configured with *k=1*, which corresponds to standard digraph convolution without higher-order proximity expansion. Under this setting, for an *L*-layer message passing model, the forward (and backward) cost per epoch is linear in the number of edges and feature dimensions, i.e., approximately $\mathcal {O}(L\cdot |E|\cdot d)$ for sparse graphs. The memory footprint is dominated by storing node features and intermediate activations, i.e., $\mathcal {O}(|V|\cdot d)$, together with $\mathcal {O}(|E|)$ for sparse adjacency and edge weights. In our setting, |*V*| is bounded by the number of distinct log templates observed within a group, and |*E*| is bounded by the group length *n*. This design keeps individual graphs relatively small and avoids the higher-order proximity expansion that may lead to quadratic memory usage in worst-case scenarios, as discussed in the original DiGCN formulation.

**Implications for online/near-real-time analysis.** Graph construction and inference are performed per log group and do not require access to the complete dataset. Therefore, graphs can be constructed and scored incrementally as groups close (e.g., by identifier completion or window expiration). Moreover, different log groups can be processed independently, enabling efficient parallelization across CPU cores or machines in practical deployments.

**Semantic embedding overhead.** The semantic node attributes are computed at the log-template level rather than per message. In practice, the embedding of each template can be precomputed once (or lazily computed upon first appearance) and cached, so the embedding cost scales with the number of unique templates rather than the total number of log messages. This significantly reduces both computation and memory overhead when processing large log volumes.

## Limitations

While the proposed framework performs strongly in our experiments, several limitations should be kept in mind.

**Grouping and graph construction.** The method assumes that log messages can be partitioned into meaningful groups and currently constructs edges by linking consecutive events. This preserves order and frequency, but does not explicitly encode richer temporal information such as durations or inter-event gaps, nor more advanced domain-specific relations among log types.

**Training-data assumptions.** Like many one-class anomaly detection methods, *Logs2Graphs* assumes access to training data that are predominantly normal. Although our contamination analysis and practical discussion help characterize this requirement, performance can still degrade when the training set is heavily polluted or when system behavior drifts substantially over time.

**Coverage of empirical comparisons.** Our benchmark prioritizes methods that are assumption-compatible and reproducible under a common protocol. As a result, it does not include supervised approaches such as LogGD, nor methods without sufficiently reproducible implementations such as GLAD-PAW. Likewise, while we motivate DiGCN as a suitable directed backbone and compare against an undirected alternative, we do not provide a full benchmark over all possible directed GNN encoders.

**Scope of explanation.** The proposed explanation mechanism is intended to identify influential log events and provide human-understandable cues for diagnosis. It should not be interpreted as causal root-cause analysis, nor as a guarantee that the highlighted nodes uniquely determine the underlying system fault.

## Conclusions

We introduced *Logs2Graphs*, a new approach for unsupervised log anomaly detection. It first converts log files to attributed, directed, and edge-weighted graphs, translating the problem to an instance of graph-level anomaly detection. Next, this problem is solved by OCDiGCN, a novel method based on graph neural networks that performs graph representation learning and graph-level anomaly detection in an end-to-end manner. Important properties of OCDiGCN include that it can deal with directed graphs and do unsupervised learning.

Extensive results on five benchmark datasets reveal that *Logs2Graphs* is at least comparable to and often outperforms state-of-the-art log anomaly detection methods such as DeepLog and LogAnomaly. Furthermore, a comparison to a similar method for graph-level anomaly detection on *undirected* graphs demonstrates that using directed log graphs leads to better detection accuracy in practice.

## Data Availability

Data is provided within online supplementary information files.

## Footnotes

1 https://github.com/ZhongLIFR/Logs2Graph.
